# Genetic Variation Analysis of the NSP1 Gene of Type 2 Porcine Reproductive and Respiratory Syndrome Virus in China

**DOI:** 10.3390/genes17080908

**Published:** 2026-07-31

**Authors:** Tianyuan Nie, Jiaman Li, Siqi Ye, Lin Wang, Ruining Wang, Xuyong Zhao, Huawei Li, Keshan Zhang, Yaqiong Ye, Mengmeng Zhao

**Affiliations:** 1 School of Animal Science and Technology, Foshan University, Foshan 528225, China; arceev587@gmail.com (T.N.); 13523782338@163.com (J.L.); 15362585374@163.com (S.Y.); zks009@126.com (K.Z.); 2 Institute of Cancer Sciences, University of Glasgow, Glasgow G12 8QQ, UK; lin.wang@glasgow.ac.uk; 3 Institute of Animal Product Quality and Safety Technology, Henan University of Animal Husbandry and Economy, Zhengzhou 450046, China; 80882@hnuahe.edu.cn (R.W.); zhaoxy6868@163.com (X.Z.); centrosome@126.com (H.L.)

**Keywords:** porcine reproductive and respiratory syndrome virus, porcine reproductive and respiratory syndrome, NSP1, genetic variation

## Abstract

**Background/Objectives:** Porcine reproductive and respiratory syndrome virus type 2 (PRRSV-2) remains the predominant genotype circulating in China and continues to evolve through mutation and recombination. Although NSP1 is recognized as a multifunctional nonstructural protein involved in immune regulation, its genetic variation among PRRSV-2 strains circulating in China has not been comprehensively characterized. **Methods:** In this study, 425 complete NSP1 sequences, primarily representing PRRSV-2 strains circulating in China, were analyzed, and 48 representative strains were selected for pairwise percent-identity visualization. **Results:** Pairwise nucleotide identity among the 48 representative strains was found to range from 80.1% to 100.0%, with the lowest value detected between AHBZ and SD-R, whereas pairwise amino-acid identity ranged from 79.6% to 100.0%, with the lowest value detected between AHBZ and HNhx. Among the 425 aligned amino-acid sequences, 290 variable sites and 93 completely conserved sites were identified. The broad-lineage distribution was composed of Lineage 1 (*n* = 79), Lineage 3 (*n* = 4), Lineage 5 (*n* = 21), and Lineage 8 (*n* = 321). A global FEL dN/dS of approximately 0.2979 was estimated from 379 non-recombinant unique haplotypes, and seven high-confidence candidate positively selected sites—42, 239, 255, 302, 367, 370, and 378—were supported by MEME, FEL, and FUBAR. A descriptive lineage-biased pattern concentrated in Lineage 5 was observed for NSP1β Val19Ile, corresponding to full-length NSP1 V199I. Eight candidate recombination events were identified using RDP4, and six representative events were further examined using SimPlot. **Conclusions:** Overall, NSP1 was found to be under predominant purifying selection while retaining localized variability, lineage-biased residue patterns, and candidate recombination signals that should be interpreted cautiously and validated experimentally.

## 1. Introduction

Porcine reproductive and respiratory syndrome (PRRS) is an acute, highly contagious disease caused by porcine reproductive and respiratory syndrome virus (PRRSV). Reproductive disorders in pregnant sows and respiratory disease in piglets and growing pigs are commonly observed [1]. Transmission is mediated mainly through contact with infected animals and contaminated materials, while continued circulation in swine populations is facilitated by persistent infection and immune modulation.

PRRSV is an enveloped, positive-sense, single-stranded RNA virus; virions are 50–65 nm in diameter and have a relatively smooth surface [2]. A genome of approximately 15 kb is contained within the virion and includes more than 10 open reading frames (ORFs). ORF1a and ORF1b are included in ORF1, which is located downstream of the 5′ untranslated region, and the nonstructural proteins NSP1–NSP8 are encoded by ORF1a [3]. In contrast to the highly conserved ORF1b, considerable size variation is observed in ORF1a, primarily because of the high variability of NSP2 [3].

PRRSV is classified into two species-level genetic groups, commonly designated PRRSV-1 and PRRSV-2. The two groups are genetically distinct, with nucleotide sequence identities of approximately 55.0–70.0% and amino-acid sequence identities of approximately 50.0–80.0% reported between representative strains [4]. PRRSV-2 is reported as the predominant group in China, where continued diversification is driven by mutation, recombination, and lineage turnover [3,5,6].

NSP1 is recognized as a multifunctional nonstructural protein and as one of the major regulators of PRRSV–host interactions. The protein is processed into NSP1α and NSP1β, and the NSP1α/NSP1β cleavage boundary at Met180/Ala181 has been verified by structural studies, with NSP1α and NSP1β corresponding to residues 1–180 and 181–383, respectively, in full-length NSP1 numbering [7,8]. Consistency of the Met180/Ala181 boundary with VR-2332 was also confirmed.

An N-terminal zinc-finger region, a papain-like cysteine protease α region, and a C-terminal extension are contained within NSP1α [7,9,10]. Roles in the antagonism of interferon-β induction have been supported for the NSP1α zinc-finger region and selected C-terminal residues by experimental studies; however, these regional findings should not be interpreted as evidence that every naturally variable residue in NSP1α has been functionally validated [7,10,11].

An N-terminal domain, a linker region, a papain-like cysteine protease β region, and a C-terminal extension are contained within NSP1β [8]. Metal-dependent nuclease-like activity has been demonstrated in the NSP1β N-terminal domain, and Lys18 and Glu32 have been identified as critical residues in that assay. By contrast, Cys90 and His159 in NSP1β local numbering (full-length NSP1 Cys270 and His339) have been identified as the catalytic residues of the papain-like cysteine protease β [8]. NSP1β Val19 has also been experimentally linked to KPNA1 degradation and interferon-activated JAK/STAT antagonism; in full-length NSP1 numbering, this position is designated residue 199 [12].

Given the immunomodulatory roles and evolutionary relevance of NSP1, genetic variation in complete PRRSV-2 NSP1 sequences was analyzed using a curated dataset of 425 sequences. Consensus-based amino-acid variability, broad-lineage distribution, candidate recombination evidence, and codon-level selection-pressure signals were evaluated, while computationally identified sites were not interpreted as experimentally validated functional determinants.

## 2. Materials and Methods

### 2.1. Materials

NSP1 records were retrieved from GenBank and curated against VR-2332. An initial dataset of 428 source records representing 426 unique GenBank accessions was obtained. Following sequence-length and quality-control screening, 425 complete NSP1 sequences were retained. Reference sequences were included for alignment, full-length residue numbering, and comparative context. Each retained NSP1 coding sequence was 1149 nt in length and encoded a 383-aa protein. Full-length NSP1 residues were numbered according to VR-2332.

### 2.2. Methods

#### 2.2.1. Representative-Strain Selection and Pairwise Identity Analysis

A 48-strain visualization set was selected to represent broad lineages, sampling years, geographic origins, and sequence diversity within the curated NSP1 dataset. For these strains, pairwise nucleotide identity was calculated across the aligned 1149-nt NSP1 coding region as the number of identical aligned nucleotide positions divided by 1149 and multiplied by 100. Pairwise amino-acid identity was calculated across the aligned 383-aa NSP1 protein as the number of identical aligned amino-acid positions divided by 383 and multiplied by 100. Lower-triangular identity matrices were subsequently generated for visualization.

#### 2.2.2. Amino-Acid Variability and Mutation Notation

Amino-acid variability was calculated from all 425 aligned NSP1 sequences, each comprising 383 aa, using consensus-based variability to describe population-level variation. Differences relative to VR-2332 were retained separately for mutation notation, such as S42P, so that reference-relative mutation frequencies were not conflated with consensus-based variability. Consensus-based variability and reference-relative mutation summaries were compiled separately.

#### 2.2.3. Phylogenetic and Broad-Lineage Analysis

A neighbor-joining (NJ) tree was generated from the 425 complete NSP1 nucleotide sequences using MEGA version 7.0 (Institute for Genomics and Evolutionary Medicine, Temple University, Philadelphia, PA, USA) [13], the p-distance model, and 1000 bootstrap replicates. Broad-lineage assignments for Lineages 1, 3, 5, and 8 were used to annotate the tree. These assignments were treated as broad-lineage categories rather than sublineage designations.

#### 2.2.4. Lineage-Distribution Analysis

For V199I and selected NSP1 amino-acid sites, amino-acid composition was summarized within each broad lineage included in this study. Statistical testing was performed only when expected-count assumptions were satisfied. Chi-square tests were applied to eligible comparisons, after which Benjamini–Hochberg false-discovery-rate correction was performed; Cramer’s V was calculated for each chi-square test. For sites 42, 239, 255, and 302, inferential testing was restricted to Lineages 1, 5, and 8 because only four Lineage 3 sequences were available and expected-count assumptions were not satisfied. V199I and sites with inadequate expected counts were interpreted descriptively.

#### 2.2.5. Recombination Evidence Review

Recombination screening in the original analysis was performed using RDP4 version 4.3 (University of Cape Town, Cape Town, South Africa) [14]. The RDP, GENECONV, BootScan, MaxChi, Chimaera, SiScan, and 3Seq methods were applied during screening. Eight candidate recombination events were contained in the available RDP4 results. Because event-level method counts and *p*-values were unavailable in the retained records, individual events could not be independently assessed against a predefined multi-method support threshold. Corresponding SimPlot profiles were available for six representative events [15].

#### 2.2.6. Selection-Pressure Analysis

The selection-pressure analysis was initiated with 425 complete NSP1 sequences. Four sequences containing ambiguous nucleotides were removed, leaving 421 strict sequences. Identical sequences were then collapsed into 387 unique haplotypes, and eight candidate recombinant strains were excluded before analysis. A final dataset of 379 non-recombinant unique haplotypes was obtained. Using the Datamonkey/HyPhy workflow (Institute for Genomics and Evolutionary Medicine, Temple University, Philadelphia, PA, USA) [16], the codon alignment was analyzed with MEME [17], FEL [18], and FUBAR [19]. MEME results were interpreted as evidence of episodic diversifying selection, whereas FEL and FUBAR results were interpreted as evidence of pervasive selection. The following thresholds were applied: *p* ≤ 0.10 for MEME, with additional summaries at *p* ≤ 0.05 and *p* ≤ 0.01; *p* ≤ 0.10 and *p* ≤ 0.05 for FEL; and a posterior probability of Pr(β > α) ≥ 0.90 for FUBAR.

#### 2.2.7. Literature-Based Descriptive Phenotype-Association Analysis

To relate the naturally variable and candidate positively selected sites to reported biological phenotypes, published PRRSV NSP1 site-directed mutagenesis, reverse-genetics, cell-based, and animal-infection studies were reviewed. Evidence was classified as direct residue-level evidence, evidence from an overlapping or adjacent functional motif, regional functional evidence, or structural-localization evidence. Reported phenotypes were grouped as interferon antagonism, KPNA1 degradation and JAK/STAT signaling, host mRNA nuclear export, viral replication, viremia, and in vivo attenuation. The resulting relationships were treated as the literature-supported descriptive associations and were not interpreted as new causal or statistical genotype–phenotype tests of the 425 GenBank-derived sequences.

## 3. Results

### 3.1. Pairwise Nucleotide Percent Identity

**Figure 1:**
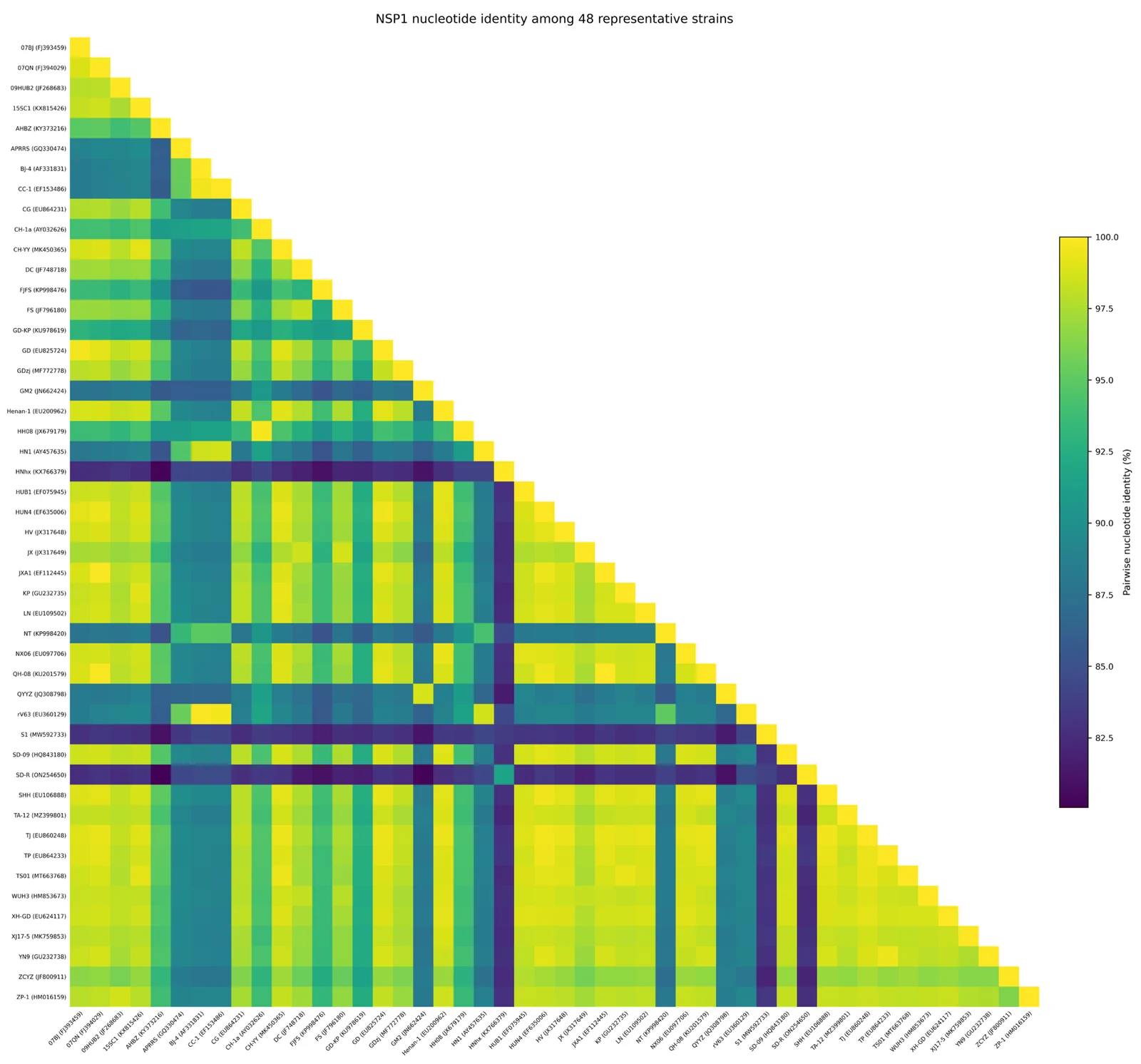
Pairwise nucleotide identity among 48 representative PRRSV-2 NSP1 sequences is shown. Percent identity was calculated across the aligned 1149-nt NSP1 coding region as the number of identical aligned nucleotide positions divided by 1149 and multiplied by 100. Rows and columns were arranged according to FASTA order, and the lower-triangular matrix, including the diagonal, was displayed without hierarchical clustering. Nucleotide percent identity is represented by color intensity. The lowest pairwise nucleotide identity in the representative set was 80.1% and was observed between AHBZ and SD-R.

The initial 428 source records and the 425 retained complete NSP1 sequences are documented in Supplementary Tables S1 and S2, respectively. Among the 48 representative NSP1 nucleotide sequences, pairwise nucleotide identity was found to range from 80.1% to 100.0% (Figure 1). The lowest identity was observed between AHBZ and SD-R, whereas the highest identity was observed between JXA1 and QH-08. The 48-strain visualization set is listed in Supplementary Table S3.

### 3.2. Pairwise Amino-Acid Percent Identity

**Figure 2:**
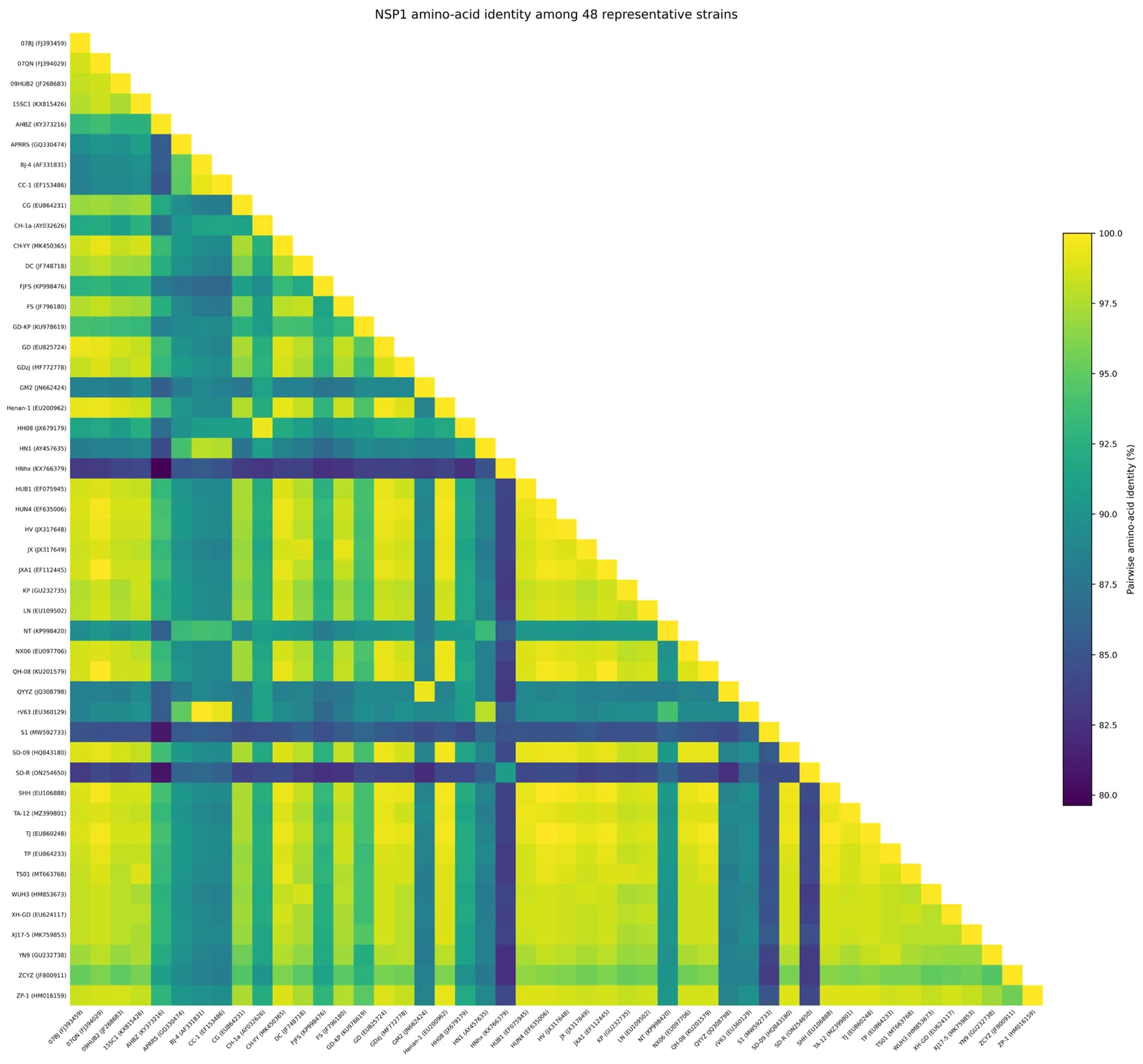
Pairwise amino-acid identity among 48 representative PRRSV-2 NSP1 sequences is shown. Percent identity was calculated across the aligned 383-aa NSP1 protein as the number of identical aligned amino-acid positions divided by 383 and multiplied by 100. Rows and columns were arranged according to FASTA order, and the lower-triangular matrix, including the diagonal, was displayed without hierarchical clustering. Amino-acid percent identity is represented by color intensity. The lowest pairwise amino-acid identity in the representative set was 79.6% and was observed between AHBZ and HNhx.

Among the 48 representative NSP1 amino-acid sequences, pairwise amino-acid identity was found to range from 79.6% to 100.0% (Figure 2). The lowest identity was observed between AHBZ and HNhx. An amino-acid identity of 100.0% was observed for seven strain pairs: 07QN/JXA1, 07QN/QH-08, BJ-4/rV63, HUN4/SHH, HUN4/TJ, JXA1/QH-08, and SHH/TJ.

### 3.3. Amino-Acid Variability and Selected Residue Numbering

**Figure 3:**
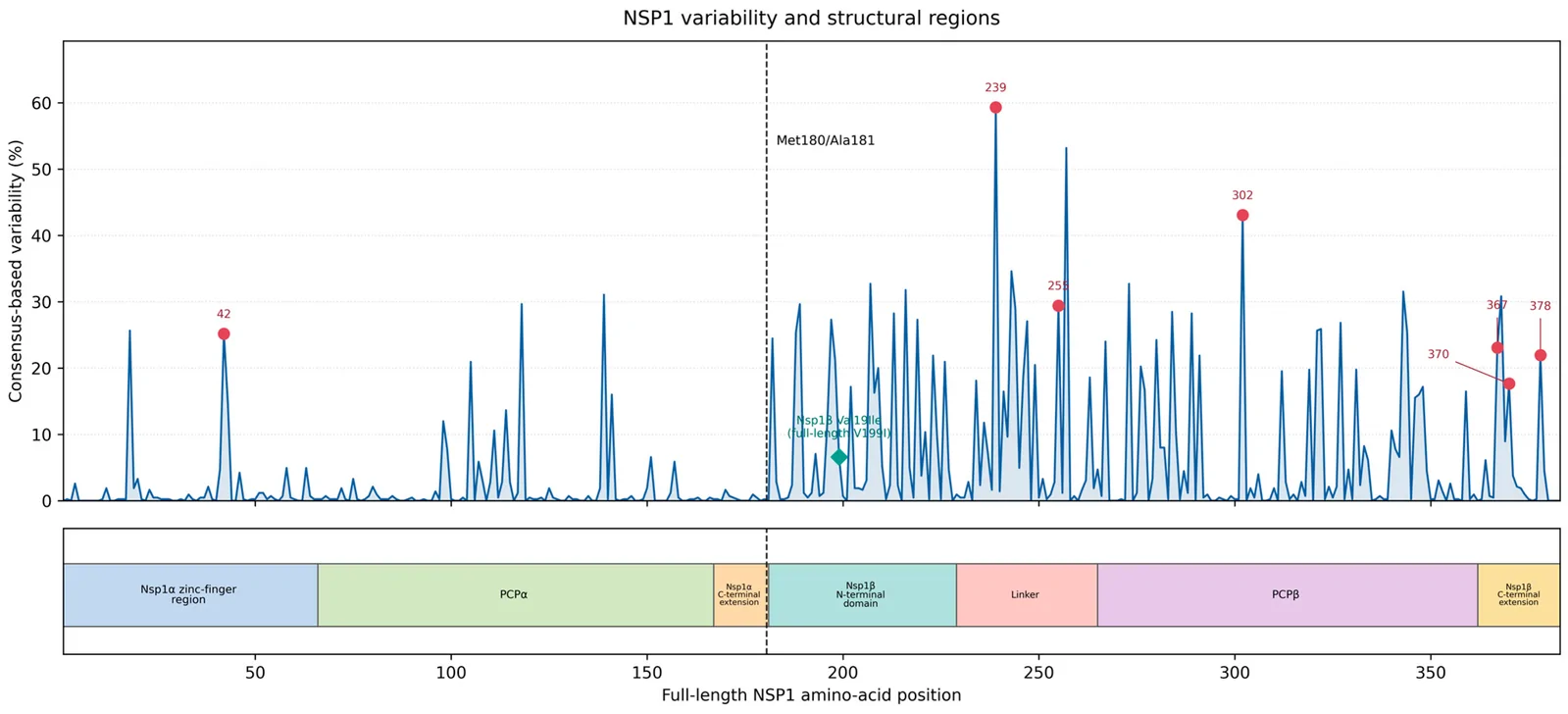
Consensus-based amino-acid variability and a structural-region map of NSP1 are shown. Variability was calculated from 425 complete aligned NSP1 amino-acid sequences and is displayed across full-length NSP1 positions 1–383. NSP1α residues 1–180 and NSP1β residues 181–383 are separated by the verified Met180/Ala181 boundary. Mutation labels, such as NSP1β Val19Ile, are reported relative to VR-2332; NSP1β Val19Ile corresponds to full-length NSP1 V199I. Sites 42, 239, 255, 302, 367, 370, and 378 are highlighted as high-confidence candidate positively selected sites supported by MEME, FEL, and FUBAR. Structural regions were assigned from published structural studies and are included for orientation. The mapped sites should not be interpreted as validated causal functional determinants.

All 425 retained NSP1 amino-acid sequences were 383 aa in length. A total of 290 variable sites and 93 completely conserved sites were identified by consensus-based analysis. Lower mean consensus-based variability was observed at full-length residues 1–113 (1.707%) than at residues 114–383 (6.284%). The NSP1β Val19Ile substitution corresponds to full-length NSP1 V199I. Within the complete 425-sequence dataset, V199I was detected in 15 sequences (3.529%), V199F in nine sequences (2.118%), and V199A in four sequences (0.941%). Detailed substitutions relative to VR-2332 and consensus-based variability values are provided in Supplementary Table S4, and the corresponding variability profile and structural-region map are shown in Figure 3.

### 3.4. Phylogenetic and Broad-Lineage Distribution

**Figure 4:**
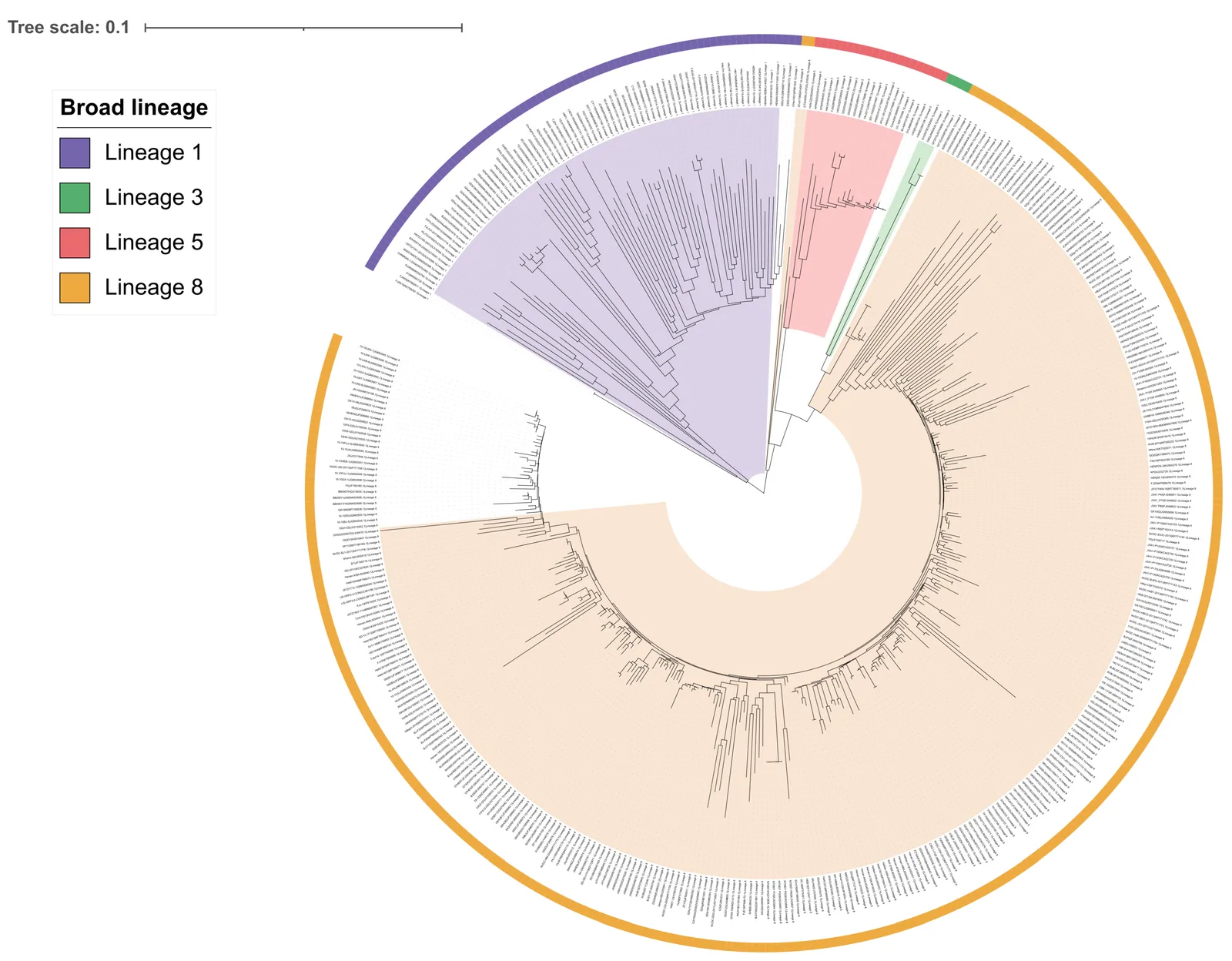
A neighbor-joining tree of 425 complete PRRSV-2 NSP1 nucleotide sequences annotated by broad lineage is shown. Broad-lineage assignments are indicated by tip colors: Lineage 1 (*n* = 79), Lineage 3 (*n* = 4), Lineage 5 (*n* = 21), and Lineage 8 (*n* = 321). These assignments should not be interpreted as fine-scale sublineage designations.

In the 425-sequence NJ tree, the retained complete NSP1 sequences were assigned to four broad-lineage groups: Lineage 1 (79 sequences), Lineage 3 (4 sequences), Lineage 5 (21 sequences), and Lineage 8 (321 sequences) (Figure 4). These broad-lineage assignments were used as the framework for the downstream lineage-distribution analysis but should not be interpreted as sublineage designations. The accession-level broad-lineage table is provided in Supplementary Table S5.

### 3.5. Lineage-Distribution Analysis

The NSP1β Val19Ile substitution, corresponding to full-length NSP1 V199I, was detected in 15 of 21 Lineage 5 sequences and was not detected in Lineages 1, 3, or 8. Because expected-count assumptions were not satisfied, this pattern was described as lineage-biased rather than as a chi-square-supported lineage association. Statistically supported lineage-associated distributions were identified for sites 42, 239, 255, and 302 among Lineages 1, 5, and 8. Lineage 3 was excluded from inferential testing because of its small sample size and inadequate expected counts. Descriptive or lineage-biased patterns were observed for sites 367, 370, and 378, but inferential testing was not performed because expected-count assumptions were not satisfied. Detailed amino-acid compositions, test decisions, Benjamini–Hochberg FDR q-values, and Cramer’s V effect sizes are provided in Supplementary Table S6.

### 3.6. Candidate Recombination Events

Eight candidate recombination events were contained in the available RDP4 results (Supplementary Figure S1). Corresponding SimPlot profiles were available for six representative events (Supplementary Figure S2). These SimPlot profiles were interpreted as representative analyses rather than as independent confirmation of all eight candidate events. Event-level RDP4 *p*-values and supporting-method counts were unavailable for reassessment and were not inferred. The event-level summary and evidence limitations are provided in Supplementary Table S7.

### 3.7. Selection-Pressure Analysis

**Figure 5:**
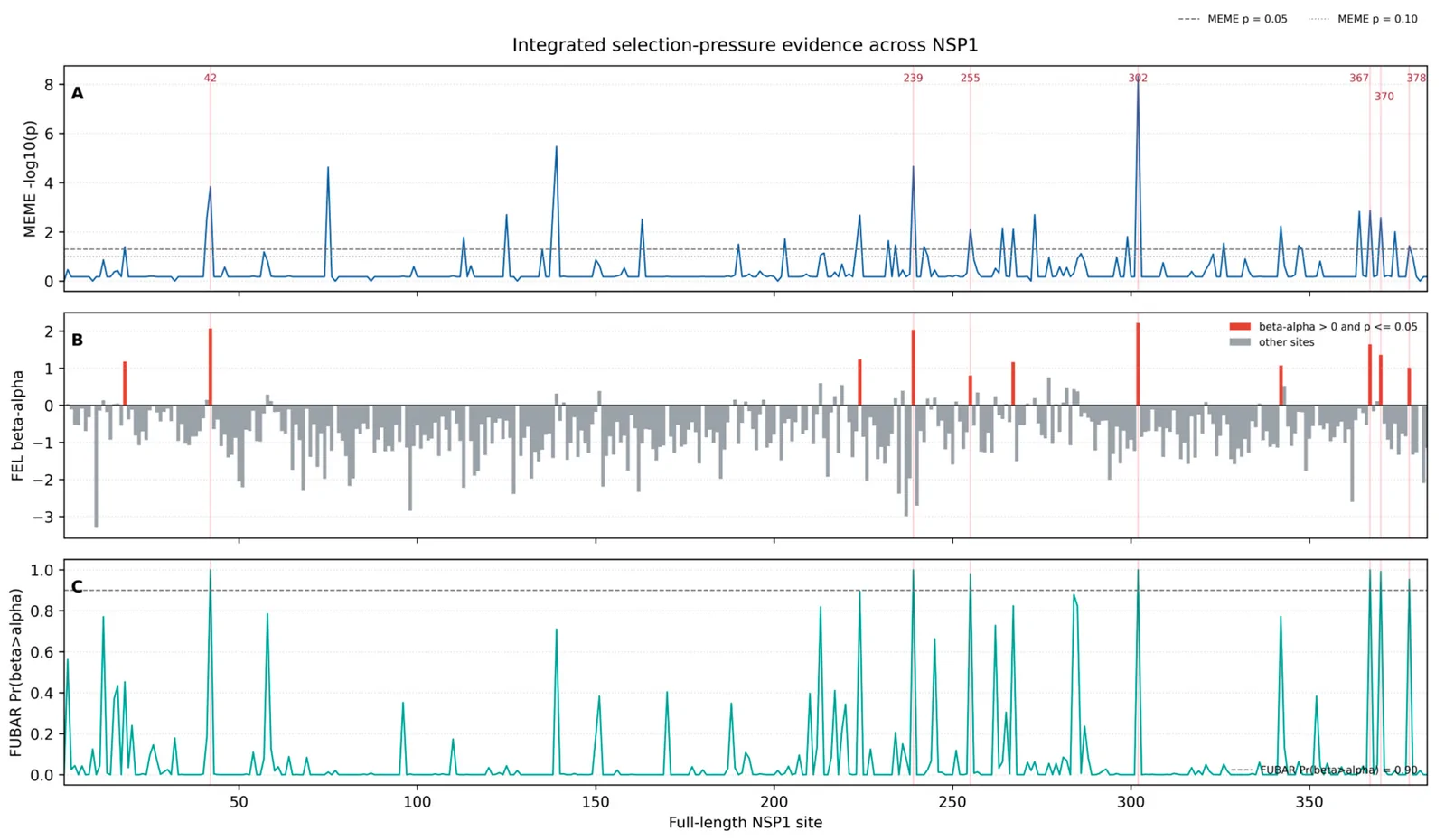
Integrated MEME, FEL, and FUBAR evidence across the 383-codon NSP1 alignment is shown for 379 non-recombinant unique haplotypes. In panel (**A**), MEME −log10(*p*) values are shown, with threshold lines at *p* = 0.05 and *p* = 0.10. In panel (**B**), FEL β − α values are shown; red bars are used to indicate β − α > 0 at *p* ≤ 0.05. In panel (**C**), FUBAR Pr(β > α) values are shown, with a posterior-probability threshold of 0.90. Sites 42, 239, 255, 302, 367, 370, and 378 are highlighted across all tracks because support was obtained from all three methods. Computational selection signals are displayed; functional effects of individual residues require experimental validation.

After preprocessing, 379 non-recombinant unique haplotypes were analyzed for selection pressure across the 383-codon NSP1 alignment. A global dN/dS of approximately 0.2979 was estimated by the FEL full model, indicating predominant purifying selection across NSP1. At *p* ≤ 0.10, 39 candidate sites were identified by MEME; 31 and 19 candidate sites were identified at *p* ≤ 0.05 and *p* ≤ 0.01, respectively. At *p* ≤ 0.10, 13 positive-selection candidates and 266 purifying-selection sites were identified by FEL, including 11 positive-selection candidates and 251 purifying-selection sites at *p* ≤ 0.05. By FUBAR, seven positive-selection candidates and 281 purifying-selection sites were identified at Pr(β > α) ≥ 0.90. Seven sites—42, 239, 255, 302, 367, 370, and 378—were supported by MEME, FEL, and FUBAR. These sites were classified as high-confidence candidate positively selected sites, although their functional effects require experimental validation (Figure 5; Supplementary Table S8).

### 3.8. Literature-Based Descriptive Phenotype Associations

A literature-based descriptive phenotype-association analysis was performed for NSP1β Val19Ile and the seven high-confidence candidate positively selected sites. Direct residue-level evidence was identified for NSP1β Val19: V19I diminished NSP1β-mediated KPNA1 degradation and inhibition of interferon-activated JAK/STAT signaling, whereas the reciprocal I19V substitution restored KPNA1 degradation [12]. Overlapping-region evidence was also identified for NSP1β residues 16–20, for which alanine substitutions increased type I interferon transcripts, attenuated viral growth in vitro, and reduced early viral growth in pigs before compensatory substitutions restored a wild-type-like phenotype [20]. For full-length site 302 (NSP1β S122), adjacent-motif evidence was provided by mutagenesis of NSP1β L126 and R128/R129, which was associated with reduced interferon suppression, restored host mRNA nuclear export, reduced viral replication, lower viremia, and attenuation of immune-suppression phenotypes [21,22]. Site 42 was supported by regional evidence from the NSP1α zinc-finger domain, whereas sites 239, 255, 367, 370, and 378 lacked direct residue-specific phenotype evidence. The evidence category, experimental system, and reported phenotype for each site are summarized in Supplementary Table S9. These literature-supported associations indicate biological plausibility but do not establish causal effects for the naturally occurring variants identified in the present sequence dataset.

## 4. Discussion

Overall conservation with heterogeneous site-level variation was indicated for PRRSV-2 NSP1 by the curated dataset. In the representative 48-strain matrix derived from 425 complete 1149-nt NSP1 coding sequences, nucleotide identity values of 80.1–100.0% and amino-acid identity values of 79.6–100.0% were observed. Within the 383-aa alignment, 290 variable sites and 93 completely conserved sites were identified. Lower consensus-based variability was observed across residues 1–113 (mean 1.707%) than across residues 114–383 (mean 6.284%), suggesting that uneven evolutionary constraints are imposed across NSP1. When these findings are considered in relation to well-recognized hypervariable PRRSV regions, NSP1 can be interpreted cautiously as being substantially constrained while still undergoing lineage- and site-specific variation.

Particular attention was given to NSP1β Val19Ile, corresponding to residue 199 in full-length NSP1 numbering. In the curated 425-sequence dataset, V199I was detected in 15 sequences (3.529%), V199F in nine sequences (2.118%), and V199A in four sequences (0.941%). V199I was concentrated in Lineage 5 (15/21 sequences) and was not observed in Lineages 1, 3, or 8. Because expected-count assumptions were inadequate for chi-square testing, this pattern is most appropriately described as lineage-biased rather than as a statistically supported lineage association. Importantly, exact-residue mutagenesis has shown that NSP1β V19I diminishes KPNA1 degradation and inhibition of interferon-activated JAK/STAT signaling, whereas reciprocal I19V restores KPNA1 degradation [12]. In addition, substitution of NSP1β residues 16–20 with alanines increased type I interferon transcripts, attenuated viral growth in vitro, and reduced early viral growth in pigs before compensatory substitutions arose [20]. Together, these studies provide direct and overlapping-region evidence linking this portion of NSP1β to interferon antagonism and replication. Nevertheless, the phenotype of each naturally occurring V199I sequence in the present dataset cannot be assumed to be identical to that of an experimentally engineered mutant.

The seven codons supported by MEME, FEL, and FUBAR were evaluated against published phenotype evidence. Site 42 is located in the NSP1α zinc-finger region, for which a role in interferon-β antagonism has been supported by regional mutagenesis evidence; however, the exact natural residue at site 42 has not been directly tested [7,10]. Full-length site 239 corresponds to NSP1β K59. Although K59A was included in an experimental nuclease-mutant panel, K59 was not identified as a critical nuclease residue; therefore, altered nuclease activity or replication should not be assigned to natural variation at this site [8]. Site 255 corresponds to NSP1β residue 75 in the linker region, for which only structural-localization evidence is currently available [8].

Full-length site 302 corresponds to NSP1β S122 and is located immediately upstream of the local 123–131 GKYLQRRLQ/SAP-like motif. Within this adjacent motif, R128A and R129A reduced NSP1β-mediated IFN-β suppression, and recombinant viruses carrying these mutations showed reduced growth, increased IFN-α and interferon-stimulated-gene responses, and lower viremia in pigs [21]. Mutation of NSP1β L126 disrupted Nup62 binding, restored host mRNA nuclear export, and reduced viral replication and titer [22]. These findings support a descriptive relationship between the region surrounding site 302 and interferon antagonism, host shutoff, replication efficiency, and in vivo viral burden. Because S122 itself has not been tested experimentally, site 302 should be interpreted as a candidate selected residue adjacent to a functionally validated motif rather than as a confirmed phenotype-determining residue. Sites 367, 370, and 378 are located in the NSP1β C-terminal extension, and no exact-residue phenotype evidence was identified for these positions. The complete evidence classification is provided in Supplementary Table S9.

A model of predominant purifying selection with localized diversifying signals was also supported by the selection-pressure analysis. A global dN/dS of approximately 0.2979 was estimated by the FEL full model, consistent with overall purifying selection across NSP1. Against this background, episodic diversifying selection was identified by MEME, whereas pervasive positive-selection candidates were identified by FEL and FUBAR. Support from all three methods was obtained for sites 42, 239, 255, 302, 367, 370, and 378, strengthening their classification as candidate positively selected sites. However, effects on replication, virulence, immune evasion, protein stability, or host adaptation cannot be demonstrated by computational selection signals alone.

The recombination evidence was interpreted conservatively. Eight candidate recombination events were indicated by the available RDP4-derived records, and corresponding SimPlot profiles were available for six representative events. Because event-level method counts, exact *p* values, and independently verified breakpoints were unavailable in the retained records, reanalysis could not be performed. Therefore, the results are best reported as eight RDP4 candidate events, of which six were further examined using representative SimPlot profiles; eight candidate recombinant sequences were excluded before selection-pressure analysis. In this way, the available evidence is preserved without overstatement of SimPlot support or implication that the original recombination analyses were rerun.

## 5. Conclusions

Based on the analysis of 425 complete PRRSV-2 NSP1 sequences, NSP1 evolution can be viewed cautiously as follows: the gene is comparatively conserved, but heterogeneous amino-acid variability, candidate recombination signals, and localized codon-level evidence of positive selection are retained. The seven sites supported by MEME, FEL, and FUBAR should be regarded as high-confidence candidate positively selected sites rather than validated functional determinants. A lineage-biased distribution concentrated in Lineage 5 was observed for NSP1β Val19Ile (full-length NSP1 V199I), but a chi-square-supported lineage association could not be established because expected-count assumptions were not satisfied. The literature-supported phenotype associations were strongest for NSP1β Val19 and the functional region adjacent to full-length site 302, linking these regions to interferon antagonism, host mRNA export, replication, and in vivo viral burden. These associations strengthen the biological relevance of the identified variation but do not establish causality for the naturally occurring variants. Further experimental studies will be required to determine whether the natural NSP1 variants affect viral replication, immune modulation, or other biological phenotypes.

## Data Availability

GenBank accession numbers and quality-control information for the source records and retained complete NSP1 sequences are provided in Supplementary Tables S1 and S2, respectively. The representative-strain set and the additional analysis outputs are provided in Supplementary Tables S3–S9 and Supplementary Figures S1 and S2.

## Supplementary Materials

The following supporting information can be downloaded at https://www.mdpi.com/article/10.3390/genes17080908/s1. Figure S1: RDP4 candidate recombination signals for eight NSP1 candidate recombinant strains/events; Figure S2: representative SimPlot profiles corresponding to six of the eight RDP4 candidate recombination events; Table S1: master list of 428 source records with quality-control status; Table S2: metadata and extraction quality control for 425 retained complete NSP1 sequences; Table S3: final 48 representative PRRSV-2 strains used for sequence-identity visualization; Table S4: amino-acid substitutions relative to VR-2332 with consensus-based variability; Table S5: broad-lineage assignments for 425 complete PRRSV-2 NSP1 sequences; Table S6: lineage-distribution statistics for V199I and selected/high-variability sites; Table S7: eight RDP4 candidate recombination events with corresponding SimPlot-profile availability and evidence limitations; Table S8: full 383-site integrated MEME, FEL, and FUBAR selection-pressure results; Table S9: literature-supported descriptive phenotype associations for NSP1β Val19Ile and the candidate positively selected sites [8,10,12,20,21,22].
